# Whole-genome resequencing shows numerous genes with nonsynonymous SNPs in the Japanese native cattle *Kuchinoshima-Ushi*

**DOI:** 10.1186/1471-2164-12-103

**Published:** 2011-02-10

**Authors:** Ryouka Kawahara-Miki, Kaoru Tsuda, Yuh Shiwa, Yuko Arai-Kichise, Takashi Matsumoto, Yu Kanesaki, Sen-ichi Oda, Shizufumi Ebihara, Shunsuke Yajima, Hirofumi Yoshikawa, Tomohiro Kono

**Affiliations:** 1 Genome Research Center, NODAI Research Institute, Tokyo University of Agriculture, 1-1-1 Sakuragaoka, Setagaya-ku, Tokyo 156-8502, Japan; 2 Graduate School of Bioagricultural Sciences, Nagoya University, Furo-cho, Chikusa-ku, Nagoya 464-8601, Japan; 3 Department of Bioscience, Tokyo University of Agriculture, 1-1-1 Sakuragaoka, Setagaya-ku, Tokyo 156-8502, Japan

## Abstract

**Background:**

Because the Japanese native cattle *Kuchinoshima-Ushi *have been isolated in a small island and their lineage has been intensely protected, it has been assumed to date that numerous and valuable genomic variations are conserved in this cattle breed.

**Results:**

In this study, we evaluated genetic features of this breed, including single nucleotide polymorphism (SNP) information, by whole-genome sequencing using a Genome Analyzer II. A total of 64.2 Gb of sequence was generated, of which 86% of the obtained reads were successfully mapped to the reference sequence (Btau 4.0) with BWA. On an average, 93% of the genome was covered by the reads and the number of mapped reads corresponded to 15.8-fold coverage across the covered region. From these data, we identified 6.3 million SNPs, of which more than 5.5 million (87%) were found to be new. Out of the SNPs annotated in the bovine sequence assembly, 20,432 were found in protein-coding regions containing 11,713 nonsynonymous SNPs in 4,643 genes. Furthermore, phylogenetic analysis using sequence data from 10 genes (more than 10 kbp) showed that *Kuchinoshima-Ushi *is clearly distinct from European domestic breeds of cattle.

**Conclusions:**

These results provide a framework for further genetic studies in the *Kuchinoshima-Ushi *population and research on functions of SNP-containing genes, which would aid in understanding the molecular basis underlying phenotypic variation of economically important traits in cattle and in improving intrinsic defects in domestic cattle breeds.

## Background

**Figure 1:**
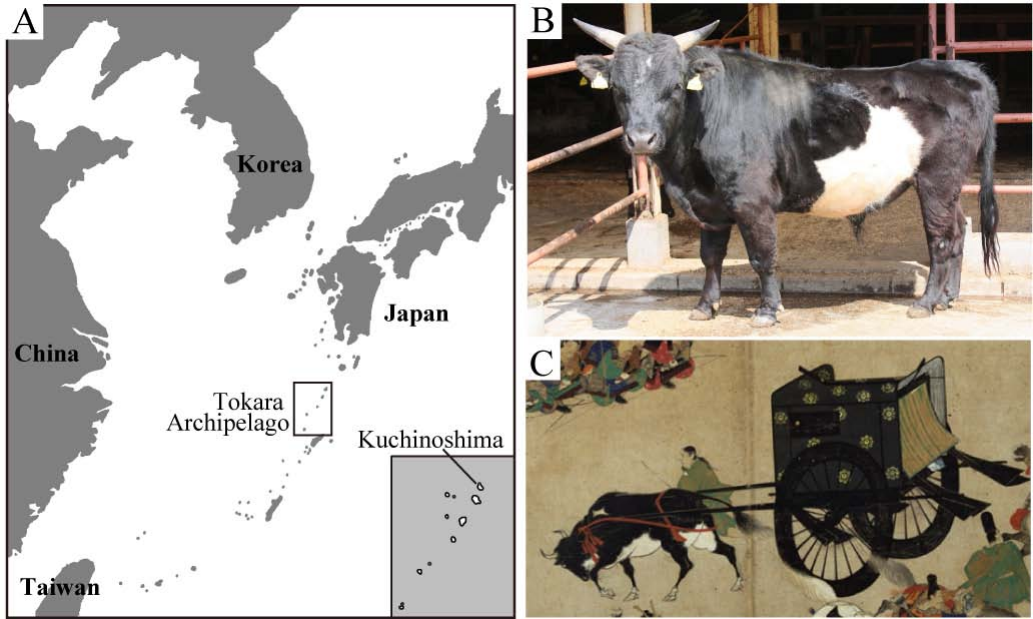
**Habitat and morphological characteristics of *Kuchinoshima-Ushi***. A: Kuchinoshima Island located in the Tokara Archipelago, Japan. B: Picture of *Kuchinoshima-Ushi *used in this study, which kept at Shitara Field, Nagoya University. C: Picture of ancient Japanese cattle pulling a traditional Japanese cart in the Heian Age. This picture scroll, entitled "Heiji-Monogatari-Emaki" and drawn in the 13th century, is a national treasure stored at the Tokyo National Museum. Image: TNM Image Archives http://TnmArchives.jp/.

The Japanese native cattle *Kuchinoshima-Ushi *has long been bred on Kuchinoshima Island in the Tokara Archipelago of Kagoshima Prefecture, Japan (Figure 1A). *Kuchinoshima-Ushi*, which have been used mainly as pack animals, are characterized genetically as being lean with a small body size (approximately 500-kg adult males and 300-kg females), wide breast, narrow waist, and horns. They have variable coat colours, including black, black with white spots, and brown. This phenotype is probably due to a population bottleneck caused by long-term isolation on this island. Similarities between ancient Japanese cattle described in historical records and *Kuchinoshima-Ushi *suggest that they have retained some features of ancient native cattle (Figure 1B, C). Currently, the four major Japanese domestic cattle breeds, namely, Japanese Black, Japanese Brown, Japanese Shorthorn, and Japanese Polled, are bred mainly for meat in Japan. These breeds were established by crossing Japanese native cattle with several European cattle breeds during the mid-19th century to improve the native stock. However, the specific characteristics inherited by modern Japanese domestic cattle are unknown.

Since the first whole-genome assembly of the human genome in 2001 [1], the sequencing and assembly of mammalian genomes have quickly progressed. The bovine genome was assembled by the international bovine whole-genome sequencing project through a combination of shotgun and bacterial artificial chromosome (BAC) sequencing of an inbred Hereford cow and her sire by using capillary sequencing [2]. Recently, Van Tassell *et al*. (2008) contributed more than 23,000 single nucleotide polymorphisms (SNPs) to the bovine SNP database (dbSNP) by next-generation sequencing using a dairy breed (Holstein) and seven major breeds of beef cattle [3], and more than 2 million SNPs have been submitted to the bovine dbSNP to date. Although the array approach with these SNPs is useful, the underlying SNP resource is far from complete for understanding genome structure [4,5]. Eck *et al*. (2009) generated 24 gigabase (Gb) of sequence with an average 7.4-fold sequence depth from a single Fleckvieh bull by next-generation sequencing [6] and identified more than 2 million previously unknown SNPs and 115,000 small insertions and deletions (indels) in comparison with the reference sequence. Although the bovine genome and HapMap projects have progressed [2,7], the sequences of Japanese cattle have not been utilized in the respective projects. Therefore, we conducted a whole-genome analysis to examine the genetic features of the Japanese native cattle *Kuchinoshima-Ushi *and to gain a better understanding of the genetic relationship between domestic cattle breeds and *Kuchinoshima-Ushi*. This study provides detailed genetic information of this breed of cattle based on 64.2 Gb of sequence data generated by next-generation sequencing.

## Results and Discussion

### Sequencing, mapping, and SNP/indel detection

**Figure 2:**
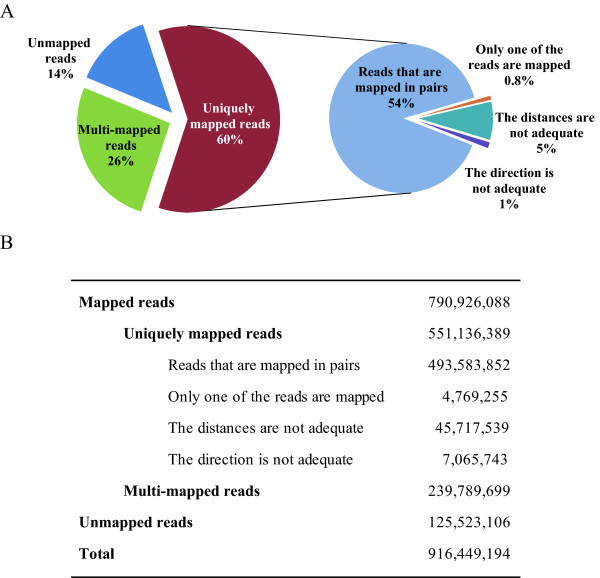
**Mapped and unmapped reads to the bovine reference genome**. Mapped reads were 790,926,088 (86%) of all the reads (916,449,194). Among the mapped reads, 239,789,699 reads (26% of the total reads) were mapped to multiple chromosomal positions and 551,136,389 reads (60% of the total reads) were uniquely mapped. The number of unmapped reads was 125,523,106 (14%).

Whole-genome sequencing was performed on a Genome Analyzer II (GAII) using the genomic DNA from a *Kuchinoshima-Ushi *male and generated 64.2 Gb of high quality sequence on 34 paired-end lanes (75-bp reads in 28 lanes and 36-bp reads in 6 lanes). Read mapping to the reference sequence (Btau 4.0) was performed using BWA [8], and 86% of the obtained reads were successfully mapped to a unique position on the bovine reference genome sequence (Btau4.0). Of the total mapped reads, 239,789,699 (26% of the total reads) were mapped to multiple chromosomal positions. We used uniquely mapped reads (551,136,389; 60% of the total reads) for further analysis (Figure 2). The chromosomal distribution of the mapped reads was unbiased except for chromosome 13 (Additional file 1). The number of reads mapped to chromosome 13 seemed higher than the average relative to the chromosome length. When we examined the read coverage of the chromosome 13 in detail, we found that most of the reads were mapped in a region known as the satellite repeat region. Although it is unknown why such a number of reads were mapped preferentially in the satellite region of chromosome 13, it is probably due to the feature of the mapping program BWA. We also performed mapping analysis using repeat-masking sequences ftp://hgdownload.cse.ucsc.edu/goldenPath/bosTau4/bigZips/, which showed that high number of reads mapped to BTA13 disappeared (Additional file 1). It shows that the region with high number of reads mapped is masked as repeat.

On an average, 93% of the genome was covered by reads (Additional file 2), and the number of mapped reads corresponds to 15.8-fold coverage across the covered region. However, the previous study describing whole-genome sequencing of a Fleckvieh bull reported that 98% of the genome was covered by the reads at a relatively low read depth (7.4-fold) [6]. The relatively lower genome coverage in our study is probably due to the fact that compared to the Fleckvieh breed, the *Kuchinoshima-Ushi *breed is evolutionarily more distantly related to the breed of the reference genome (Hereford). The difference in coverage can also be due to the number of prepared libraries (three different libraries in the Fleckvieh breed, whereas only one library in the *Kuchinoshima-Ushi *breed).

**Figure 3:**
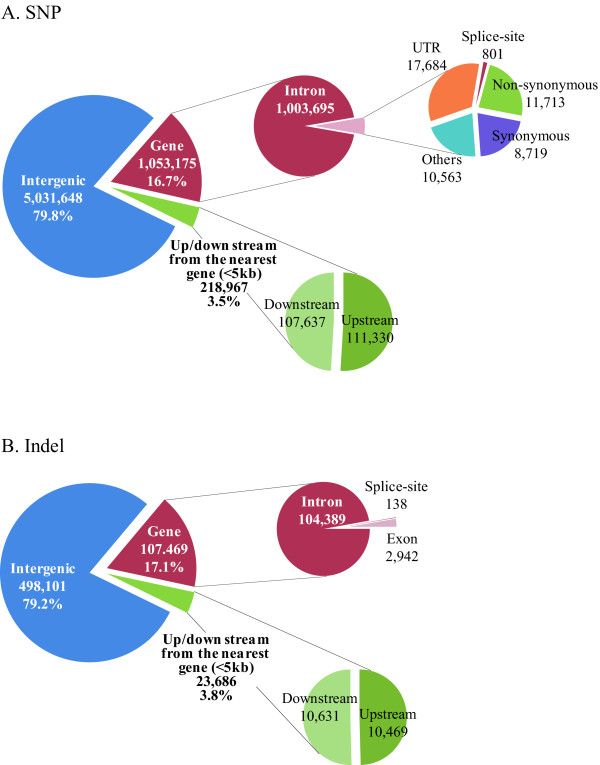
**Identified SNPs and small indels**. Identified SNPs (A) and small indels (B). We used the RefSeq and GenBank gene sets (16,635 genes) to annotate the detected variants. We found 1,003,695 intron SNPs, 17,684 untranslated regions (UTRs), 801 splice-site SNPs, 8,719 synonymous SNPs, and 11,713 nonsynonymous substitutions. Among the identified indels, 104,389 were found in intron regions, 2,942 in exon regions, and 138 in splice-sites.

Using SAMtools [9], 6,303,790 SNPs were detected; 3,491,313 (55.4%) were heterozygous and 2,812,477 (44.6%) were homozygous. Of the identified SNPs, 5,031,648 (79.8%) were located in intergenic regions and 218,967 (3.5%) were within the 5-kb regions upstream or downstream from gene regions (Figure 3A). The remaining SNPs (1,053,175; 16.7%) were located in gene regions (Figure 3A). The number of SNPs in each chromosome decreased in accordance with the decrease of the length of chromosomes, and the SNP density in each 1-kb region of the total genome sequence showed that the chromosomal distribution of the SNPs was unbiased (Additional file 3, 4). The locations of the identified SNPs were also compared with those already published (dbSNP Build 129; latest version of dbSNP data ftp://ftp.ncbi.nlm.nih.gov/snp/organisms/cow_9913/chr_rpts/); 12.9% of the SNP sites were found in the database, and the remaining 87.1% were new. The percentage of newly identified SNPs in this study closely agreed with the results from a previous study [6]. We also investigated indel events and found 629,256 (284,007 insertions and 345,249 deletions). Of these indels, 498,101 (79.2%) were in intergenic regions, 23,686 (3.8%) were within 5-kb upstream or downstream of gene regions, and 107,469 (17.1%) were in gene regions (Figure 3B). The number of indels in each chromosome decreased in accordance with decrease of the length of the chromosomes (Additional file 3). The length of most indels was 1 bp (Additional file 5).

Sequence data were deposited in the DDBJ Read Archive (DRA) [Accession #: DRA000172] and identified SNPs excluding ambiguous bases and those mapping to multiple locations unless providing more than 5 kbp flanking sequences were submitted to the single nucleotide polymorphism database (dbSNP) at NCBI under the handle TUANGRC. In addition to submitting data to the standard repositories, the positions of the SNPs for *Kuchinoshima-Ushi *can be viewed in a customized installation of the UCSC Genome Browser http://www.nodai-genome.org/btau/cgi-bin/hgGateway, along with supporting evidence (the number of reads for each allele and the density of the SNPs).

### Functional annotation of nonsynonymous SNPs (nsSNPs) and nsSNP-containing genes

The SNPs in gene regions were annotated using the RefSeq and GenBank gene sets (16,635 genes). We found 1,004,496 SNPs in introns, 17,684 in untranslated regions (UTRs), 801 SNPs in splice-sites, and 20,432 coding SNPs leading to 11,713 nonsynonymous nucleotide substitutions (Figure 3A). The percentage of nonsynonymous changes in the coding region was 57.3%, which was higher than that of any other previous studies of whole-genome resequencing in humans and cattle [6,10-15]. This finding would indicate on a possible occurrence of pseudogenes, the existence of proteins whose functions have been severely affected by extensive amino acid substitution, or significant differences in segmental duplications or copy number variants. Alternatively, the number of nsSNPs might have increased due to the false positives created by incomplete annotation information.

**Figure 4:**
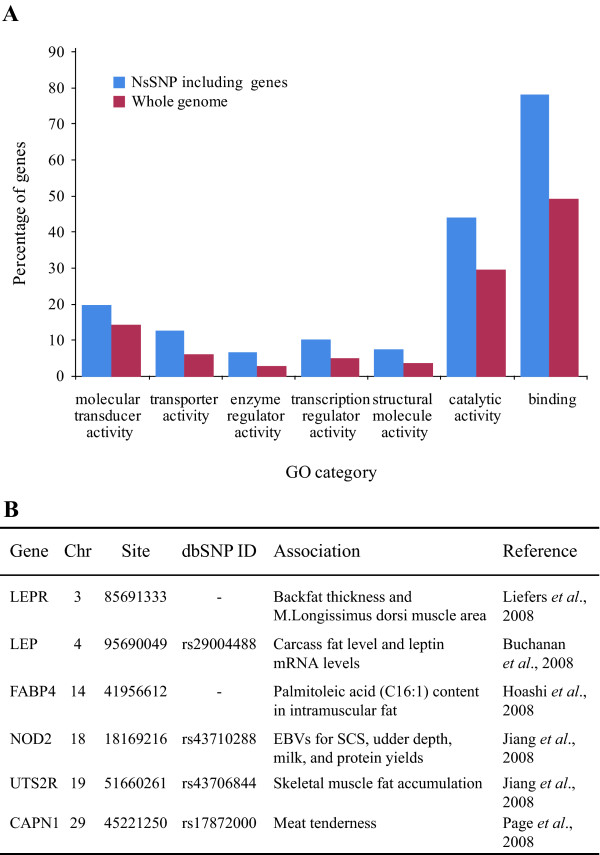
**Functional annotation of the genes containing nsSNPs**. A. Gene ontology (GO) terms enriched in the 100 genes containing the highest number of nsSNPs. Blue columns show the percentage of genes among these nsSNP-containing genes, and red columns show the percentage of genes within the whole genome. In this chart, the secondary level terms are used as GO terms. B. Identified nsSNPs reportedly associated with phenotypes in the other breed of cattle. Six SNP sites exactly matched mutations reported in previous studies to be associated with economically important traits [25,26,29,30,35]. "-" implies the absence of record in the dbSNP database. EBV: estimated breeding values; SCS: somatic cell score.

In our *Kuchinoshima-Ushi *data set, nsSNPs were detected in a total of 4,643 genes, which are listed in Additional file 6. Gene ontology (GO) terms associated with the 100 genes containing the highest number of nsSNPs were compared to those of all genes in the bovine whole genome by using the web-based tool agriGO [16,17]. The analysis showed that the genes associated with molecular functions such as protein binding, catalytic activity, and metabolic pathways and their regulation were enriched among the top 100 nsSNP-containing genes in the *Kuchinoshima-Ushi *population (Figure 4A, Additional file 7). These results suggest the possibility that phenotypes associated with these genes may represent specific characteristics of the *Kuchinoshima-Ushi *breed. In contrast, genes involved in environmental adaptation such as sensory perception or immune function were not enriched in the list of the top 100 nsSNP containing genes.

We also examined whether nsSNP-containing genes were reportedly associated with economically important traits such as meat/milk production, growth rate, and domestication in other breeds of cattle. In order to extract information from studies reporting relationships between SNPs/genes and traits, we performed a PubMed search using economical traits such as growth rate or meat production as the query and limited the search to studies of *Bos Taurus*. From the result of the search, GenBank/EMBL/DDBJ nucleotide records reported in the obtained articles as well as sequences referred to in the articles as references (RefSeqs) were extracted and compared with the list of nsSNP-containing genes in our analysis of *Kuchinoshima-Ushi*.

Of the 4,643 nsSNP-containing genes, 708 matched to genes that have been reported to be potentially associated with economically important traits such as meat/milk production, growth rate, and domestication in other cattle breeds [18-22] (the list of genes containing nsSNPs that were reported as trait-associated in other cattle breeds is shown in Additional file 8). Some of the genes were also found to reside at positions of significant quantitative trait loci (QTL; data from Cattle QTLdb http://www.animalgenome.org/cgi-bin/QTLdb/BT/index); for example, body growth-associated genes such as genes for growth hormone (*GH*), growth hormone receptor (*GHR*), and leptin receptor (*LEPR*) [18-20]; lactation-related genes like prolactin receptor (*PRLR*) and caseins (*CSN*); and genes encoding immune-related proteins in milk, such as toll-like receptors (*TLR*s) [21,22].

Recently, associations between SNPs and phenotypes have been reported in many studies, including cattle [23,24]. Some SNP sites identified in *Kuchinoshima-Ushi *have already been reported to be associated with phenotypes in other cattle breeds (Figure 4B). For example, a mutation found in the fatty acid binding protein 4 (*FABP4*) was reported to be associated with palmitoleic acid (C16:1) content in intramuscular fat in Japanese Black cattle [25]. A mutation of the urotensin 2 receptor (*UTS2R*) was associated with skeletal muscle fat accumulation in Wagyu × Limousin population [26] and a mutation in calpain 1 (*CAPN1*) was associated with meat tenderness in Aberdeen Angus-cross cattle [27]. The nsSNPs found in the nucleotide-binding oligomerization domain containing 2 (*NOD2/CARD15*) gene were associated with the estimated breeding values for somatic cell score in Canadian Holstein cattle [28]. Identified SNP sites in the *LEP *and *LEPR *genes were associated with fat content in carcass meat of other cattle breeds including Nellore, Holstein-Friesian, Angus, Charolais, Hereford, and Simmental [19,29,30].

Nevertheless, population genetic studies in the *Kuchinoshima-Ushi *breed are required to validate the identified nsSNPs and to examine their association with relevant phenotypic traits in this population. Also, most nsSNPs identified in *Kuchinoshima-Ushi *have yet to be reported, nor have their functions or associations been investigated; new nsSNPs identified in this study will be valuable for future research.

### Phylogeny of bovine-related species

**Figure 5:**
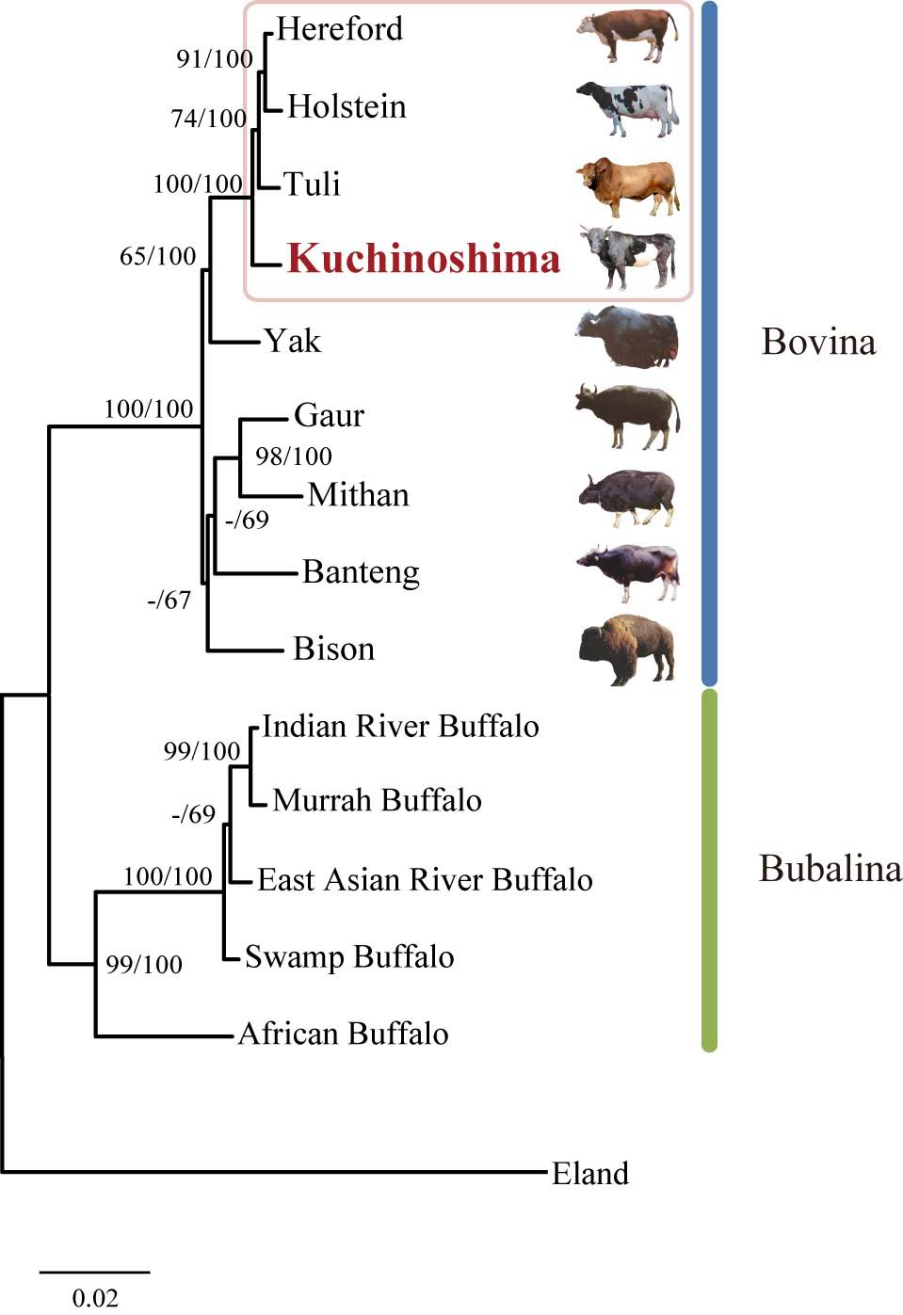
**Maximum likelihood tree of bovine-related species**. Breeds of the species "*Bos Taurus*" (Hereford, Holstein, Tuli, and *Kuchinoshima-Ushi*) were boxed in pink. Numbers beside internal branches indicate bootstrap (BS) values (> 50%) from 1,000 replicates (left) and Bayesian posterior probabilities (right), respectively (shown as percentages). "-" indicates a node not recovered in the Bayesian analysis or <50% of BS values. "Mithan", also called "gayal", is a domesticated gaur.

To understand the genetic relationships between *Kuchinoshima-Ushi *and other breeds of cattle, we carried out a phylogenetic analysis. The resultant maximum likelihood tree of bovine-related species is shown in Figure 5. Bovinae subfamily relationships were largely consistent with those in an earlier study [31], with the exception of the phylogenetic position of bison and the sister-group relationship between East Asian river buffalo (*Bubalus bubalis*) and swamp buffalo (*Bubalus carabasis*). Two subtribes, namely, Bovina and Bubalina, formed a monophyletic group with high maximum likelihood (ML) bootstrap (BS) and Bayesian posterior probability (PP) support (BS > 99%, PP = 100%), respectively. In the Bovina clade, Hereford, Holstein, Tuli, and *Kuchinoshima-Ushi *comprised a monophyletic group with high statistical support (BS = 100%, PP = 100%), and *Kuchinoshima-Ushi *was the sister lineage of all other breeds of *Bos taurus *cattle with relatively high statistical support (BS = 74%, PP = 100%). Among species of wild cattle that have never been subjected to selective breeding, such as Banteng and Gaur, *Kuchinoshima-Ushi *was the nearest species of domesticated cattle. These data also suggest that *Kuchinoshima-Ushi *would be an appropriate breed for investigating adaptations associated with the domestication of wild cattle. When the sequence data of Japanese domestic breed of cattle such as Japanese Black will be available, the phylogenetic position of "Japanese native cattle" should be revealed more clearly.

## Conclusions

In the present study, Japanese native cattle *Kuchinoshima-Ushi*, which have retained their phenotype over time without being affected genetically by European domestic breeds, were used for whole-genome resequencing with a next-generation sequencer. We identified 6.3 million SNPs, of which more than 5.5 million (87%) were found to be new. Among the identified SNPs, some nonsynonymous SNPs were found in genes that have been reported as candidate genes for QTL affecting economically important traits in other cattle breeds. The results of phylogenetic analysis showed the phylogenetic position of *Kuchinoshima-Ushi*. In the current status of domestic cattle, repeated selection for specific traits such as weight gain as well as milk and meat production has progressed, but problems have emerged with unconsidered traits such as reproduction and disease resistance. Our results provide a framework for future investigations aiming at the understanding of gene functions and identifying the molecular basis underlying phenotypic variation of economically important traits in domestic cattle breeds. It would contribute to the development of a new, more efficient breeding system preferentially for East Asian domestic cattle breeds including Japanese one. Furthermore, genome information for *Kuchinoshima-Ushi *is expected to be important not only in revealing the genetic structure of a geographically isolated breed originating from ancient, native Japanese cattle but also in identifying genetic traits that distinguish domestic breeds from original, undomesticated species.

## Methods

### Breeding conditions for *Kuchinoshima-Ushi*

For our experiments we used genomic DNA from a horned 119-month-old male *Kuchinoshima-Ushi *(#8915) (Figure 1B) that weighed 485 kg and was black with large white spots. The cattle in the wide field of Nagoya University are bred under good and relaxed conditions. In 1990, one male and three female *Kuchinoshima-Ushi *were captured on Kuchinoshima Island and transferred to the stock farm at Shitara Field, Nagoya University. In 1993, two males and three females were brought to the field from the Nagoya Animal Science Foundation. By 2004, the group had increased to 23 males and 22 females with inbred conditions in a closed colony.

### DNA library construction and sequencing

Blood sampling was carried out according to the 'Regulations for Animal Experiments in Nagoya University' and the 'Guidelines for the Care and Use of Laboratory Animals', as specified by the Tokyo University of Agriculture.

Genomic DNA was extracted from blood using standard phenol/chloroform extraction methods [32]. A genomic DNA library was prepared using a paired-end DNA sample prep kit (Illumina Inc., San Diego, CA, USA) according to the manufacturer's instructions (Preparing Samples for Sequencing Genomic DNA, Part # 1003806 Rev.B, Illumina) with slight modification as follows: 2 μg of DNA were fragmented to a median fragment size of 200 bp using Adaptive Focused Acoustics (Covaris, Inc., Woburn, MA, USA). After size selection on a 2% agarose gel, 10 μl of the DNA fragments was enriched by 12 cycles of PCR. For quality control, an aliquot of the library was cloned into a sequencing vector (TOPO TA Cloning^® ^Kit for Sequencing, Invitrogen Corp., Carlsbad, CA, USA) and 96 clones were sequenced by sanger sequencing. We found that all sequences were unique, and duplicates were not detected in the analyzed sample. BLAST search in the bovine genome sequence database using these sequences revealed high (90%) similarities to respective bovine sequences. DNA concentration was measured using UV-1800 UV-VIS Spectrophotometer (Shimadzu Corporation, Kyoto, Japan). The genomic DNA library was diluted to 10 nM, and a 2 μl aliquot was used to generate clusters on the Illumina Cluster Station using the Paired-End Cluster Generation Kit v2 (Illumina) and sequenced on the GAII with the SBS 36-cycle Sequencing Kit v3 following the manufacturer's instructions (Using the Paired-End Cluster Generation Kit v2 on the Cluster Station and Paired-End Module, Part #: 1005629 Rev.C; Using the SBS Sequencing Kit v3 on the Genome Analyzer, Part #: 1005637 Rev.A, Illumina) as 75-bp and 36-bp reads (28 and 6 paired-end lanes, respectively).

### Software

For alignment and annotation of the sequence reads, we used the bovine genome assembly Btau 4.0 ftp://hgdownload.cse.ucsc.edu/goldenPath/bosTau4/chromosomes/ as a reference source. In this study, sequence scaffolds not yet assigned to specific chromosomes were excluded and no repeat masker was applied to the assembly.

Image analysis and ELAND alignment were performed with Illumina's Pipeline Analysis software ver. 1.4. Sequences passing through the standard Illumina GA pipeline filters (i.e., clusters with intensities greater than 0.6-times the average of the highest and the sum of the two highest intensities for the first 25 cycles) were retained for further analysis.

For short-read alignment and consensus assembly, we used a recently developed algorithm, BWA ver. 0.5.0 (the outline of the algorithm is available online http://bio-bwa.sourceforge.net/) [8]. The BWA default values for mapping were as follows: maximum edit distance (maxDiff) = 0.04, maximum number of gap opens (maxGapO) = 1, maximum number of gap extensions (maxGapE) = -1, disallow a long deletion within bp (nDelTail) = 16, disallow an indel within bp (nIndelEnd) = 5, take the first subsequence as seed (seedLen) = infinite, maximum edit distance in the seed (maxSeedDiff) = 2, number of threads (nThrds) = 1, mismatch penalty (misMsc) = 3, gap open penalty (gapOsc) = 11, gap extension penalty (gapEsc) = 4, and parameter for read trimming (trimQual) = 0. After read mapping, we discarded the reads mapped to multiple chromosomal positions and unmapped reads. Only reads mapped to a unique position on the reference genome sequence were used for SNP calling.

To call SNPs, we used SAMtools [9] and applied additional filters as follows: minimum read depth = 3, minimum read depth calling the SNP = 2, and a 30% cutoff of percent aligned reads calling the SNP per total mapped reads at the SNP sites. We also filtered these identified SNPs with more stringent parameters (i.e., minimum depth = 4, minimum SNP = 2, and 30% or higher aligned reads calling the SNP; and minimum depth = 5, minimum SNP = 3, and 30% or higher aligned reads calling the SNP), but the difference in the number of SNPs was small (Additional file 9). Heterozygous and homozygous SNPs were distinguished using an 80% cutoff of percent aligned reads calling the SNP. We also used BWA to estimate the sequence read depth, which influences the coverage and accuracy of SNP calling. After SNP calling, we annotated the SNPs using the GenBank and RefSeq gene sets (16,635 genes; the gene set is available from the UCSC download site ftp://hgdownload.cse.ucsc.edu/goldenPath/bosTau4/database).

### Phylogenetic analysis

We used 15 species from the Bovinae subfamily (9 species from subtribe Bovina, 5 species from subtribe Bubalina, and one species from tribe Tragelaphini as an outgroup) for the phylogenetic analysis (Additional file 10). On the basis of a previous study [31], we used 10 nuclear genes (listed in Additional file 11). All of the sequence data except for that of *Kuchinoshima-Ushi *were previously determined and published (accession numbers can be seen in Additional file 11). Sequences of the genes from *Kuchinoshima-Ushi *were determined reflecting its SNP information in the reference sequence data. We concatenated newly-determined sequences of *Kuchinoshima-Ushi *and the published sequences and aligned them for the phylogenetic analysis. Phylogenetic trees were estimated using partitioned ML and partitioned Bayesian methods using RAxML ver. 7.0.3 [33] and MrBayes ver. 3.1.2 [34], respectively.

## Authors' contributions

SY, HY, and TK conceived of the study and participated in its design and coordination. SO and SE kept *Kuchinoshima-Ushi *and provided samples required for sequencing. RK, KT, YS, YK, TM, and YK performed the sample preparation and the sequencing experiments. RK, KT, and YS performed the data analysis. RK, KT, and TK drafted the manuscript. SY, HY, TK, SO, and SE supervised research and all authors contributed to and approved the final manuscript.

## Supplementary Material

Additional file 1 **Reads for all chromosomes of repeat masked and unmasked genome assembly**. In each chromosome, left columns show the reads mapped to the assembly without repeat masking and right columns show those to the repeat-masked assembly. Among the reads that were mapped to the reference genome sequence, most were mapped in pairs (blue column in each chromosome). However, in some read pairs, only one was mapped (red column). Additionally, some read pairs were mapped, but the distances or directions were not adequate (green columns). Length of the chromosomes is shown in the yellow line. High number of reads mapped to BTA13 of the assembly without repeat masking (left column) was removed in the repeat-masked assembly (right column). Click here for file

Additional file 2 **Length of the regions that are covered by reads for each chromosome**. Length of the regions that are covered by reads for each chromosome. The length of chromosomes is shown in the blue columns, and that of the regions covered by reads is shown in the red columns. The percentage of the regions that are covered by reads in each chromosome is indicated by the green line. On an average, 93% of the genome is covered by reads. Click here for file

Additional file 3 **The number of identified SNPs and indels for each chromosome**. SNPs are shown in the blue columns, and indels are shown in the red columns. Length of chromosomes is indicated by the green line. Click here for file

Additional file 4 **SNP distribution on each chromosome**. SNP density (SNPs per 1 kbp) is plotted by physical position. Relative length of the chromosomes was correlated with the length of each chromosome without repeat regions. Click here for file

Additional file 5 **Distribution of the size of indels**. We identified 284,007 insertions (positive values) and 345,249 deletions (negative values). Click here for file

Additional file 6 **The list of genes containing nsSNPs**. The list of genes containing nsSNPs along with the accession numbers (ss number). Click here for file

Additional file 7 **GO terms which were over-represented in nsSNP-containing genes**. Click here for file

Additional file 8 **The list of genes containing nsSNPs that were reported as trait-associated in other cattle breeds**. Click here for file

Additional file 9 **The number of SNPs and indels with various filters**. Detected SNPs and indels were filtered with additional filters and the number of homozygous and heterozygous SNPs and indels was compared. Parameters for the filters were (1) Depth: the number of reads mapped to the SNP sites, (2) SNPs: the number of reads calling SNP at the SNP site, and (3) Mutation: the cutoff value of percent aligned reads calling the SNP per total mapped reads at the SNP sites. Cut off value was 30% in all filters. In the table, "%" means the reduced percentage of the number of SNPs/indels compared with basic parameters (i.e., Depth≥ 3, SNPs≥ 2, and Mutation≥ 30). Click here for file

Additional file 10 **List of species examined in this study**. Click here for file

Additional file 11 **Summary of the genes sequenced for the phylogenetic reconstruction**. List of the genes and their accession numbers which were used for the phylogenetic analysis. Click here for file
