# Supplementary figures and images for: Whole-genome resequencing shows numerous genes with nonsynonymous SNPs in the Japanese native cattle Kuchinoshima-Ushi
Source: BMC Genomics. 2011 Feb 10;12:103. doi: 10.1186/1471-2164-12-103 (PMC3048544; doi:10.1186/1471-2164-12-103)

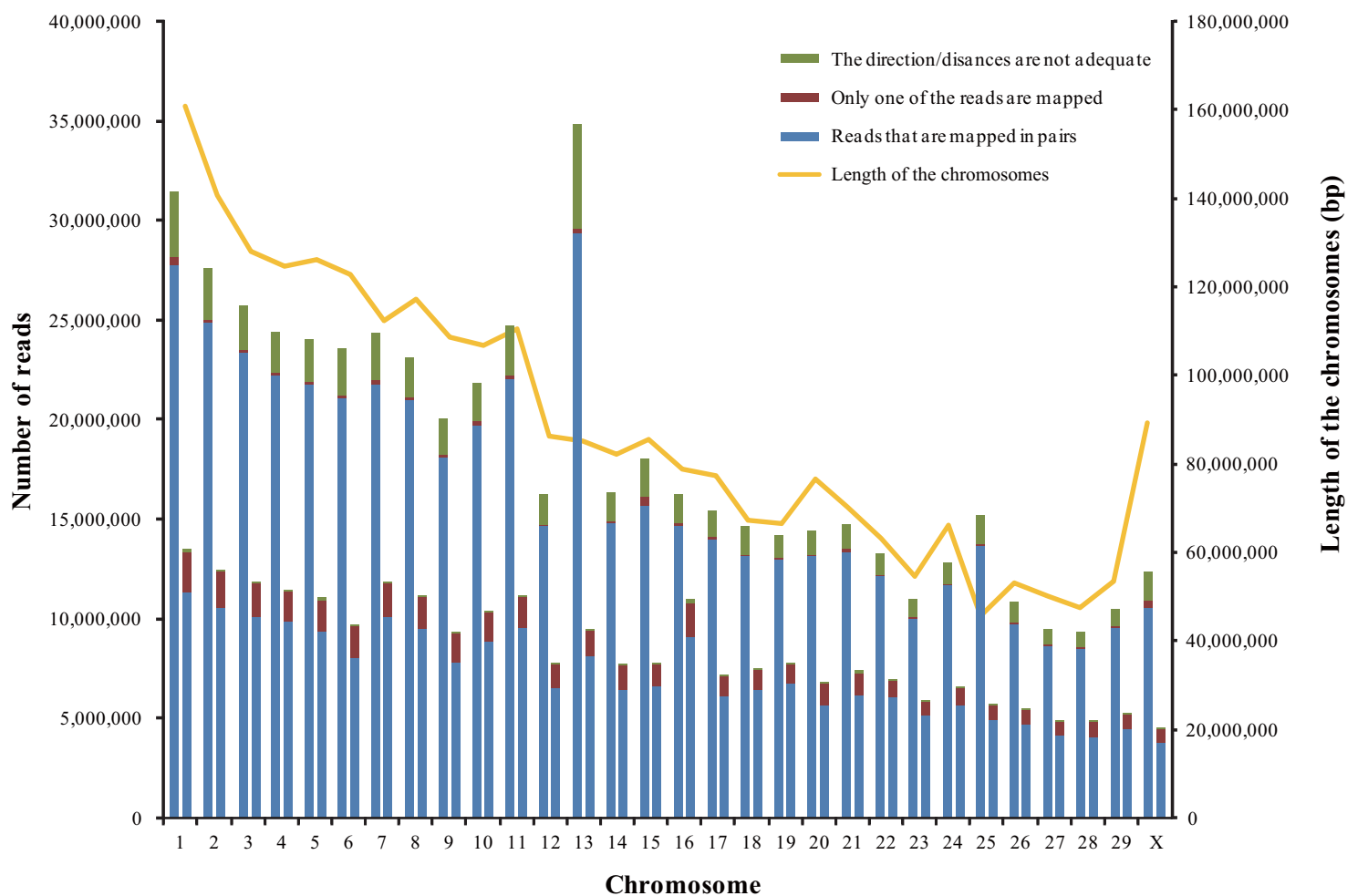

Supplement: Additional file 1 — Reads for all chromosomes of repeat masked and unmasked genome assembly. In each chromosome, left columns show the reads mapped to the assembly without repeat masking and right columns show those to the repeat-masked assembly. Among the reads that were mapped to the reference genome sequence, most were mapped in pairs (blue column in each chromosome). However, in some read pairs, only one was mapped (red column). Additionally, some read pairs were mapped, but the distances or directions were not adequate (green columns). Length of the chromosomes is shown in the yellow line. High number of reads mapped to BTA13 of the assembly without repeat masking (left column) was removed in the repeat-masked assembly (right column). [file 1471-2164-12-103-S1.PDF]

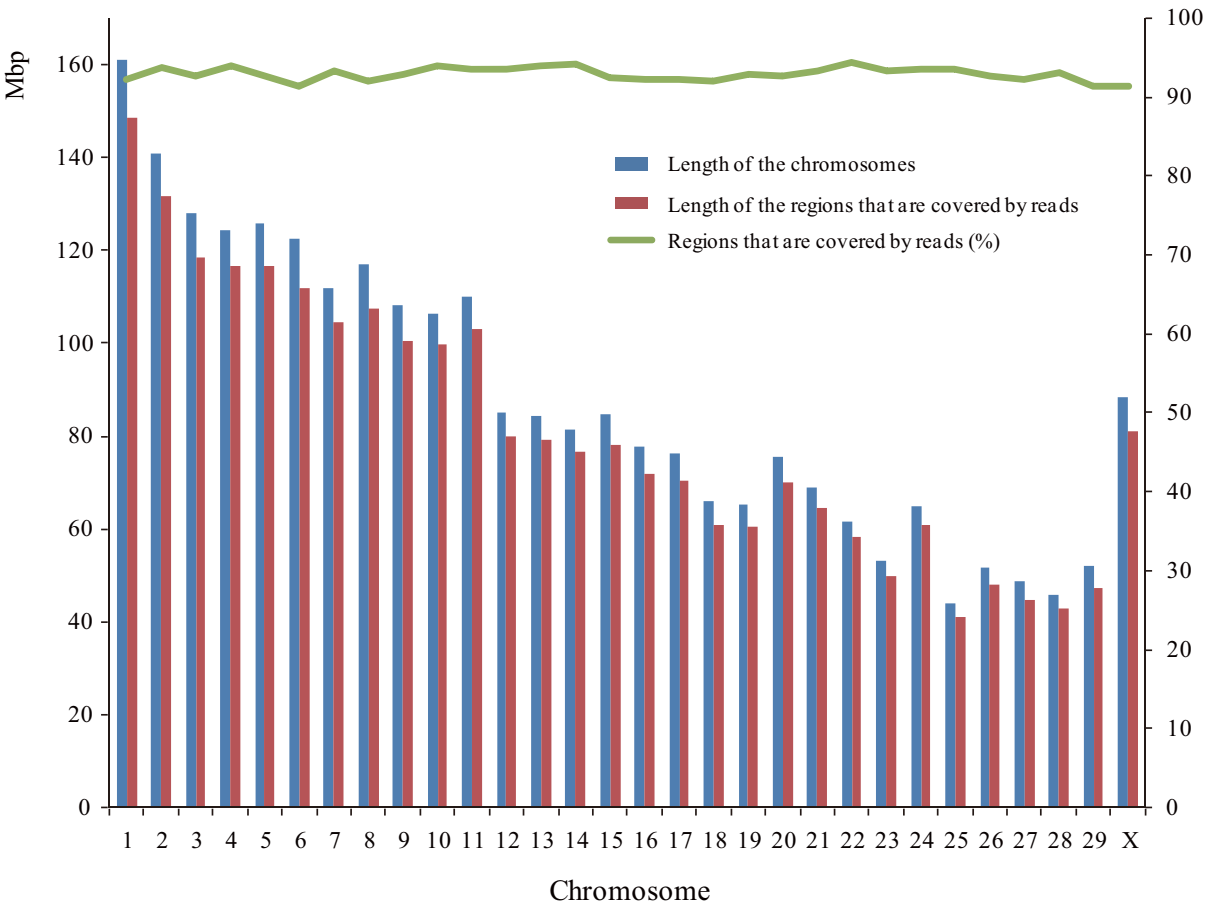

Supplement: Additional file 2 — Length of the regions that are covered by reads for each chromosome. Length of the regions that are covered by reads for each chromosome. The length of chromosomes is shown in the blue columns, and that of the regions covered by reads is shown in the red columns. The percentage of the regions that are covered by reads in each chromosome is indicated by the green line. On an average, 93% of the genome is covered by reads. [file 1471-2164-12-103-S2.PDF]

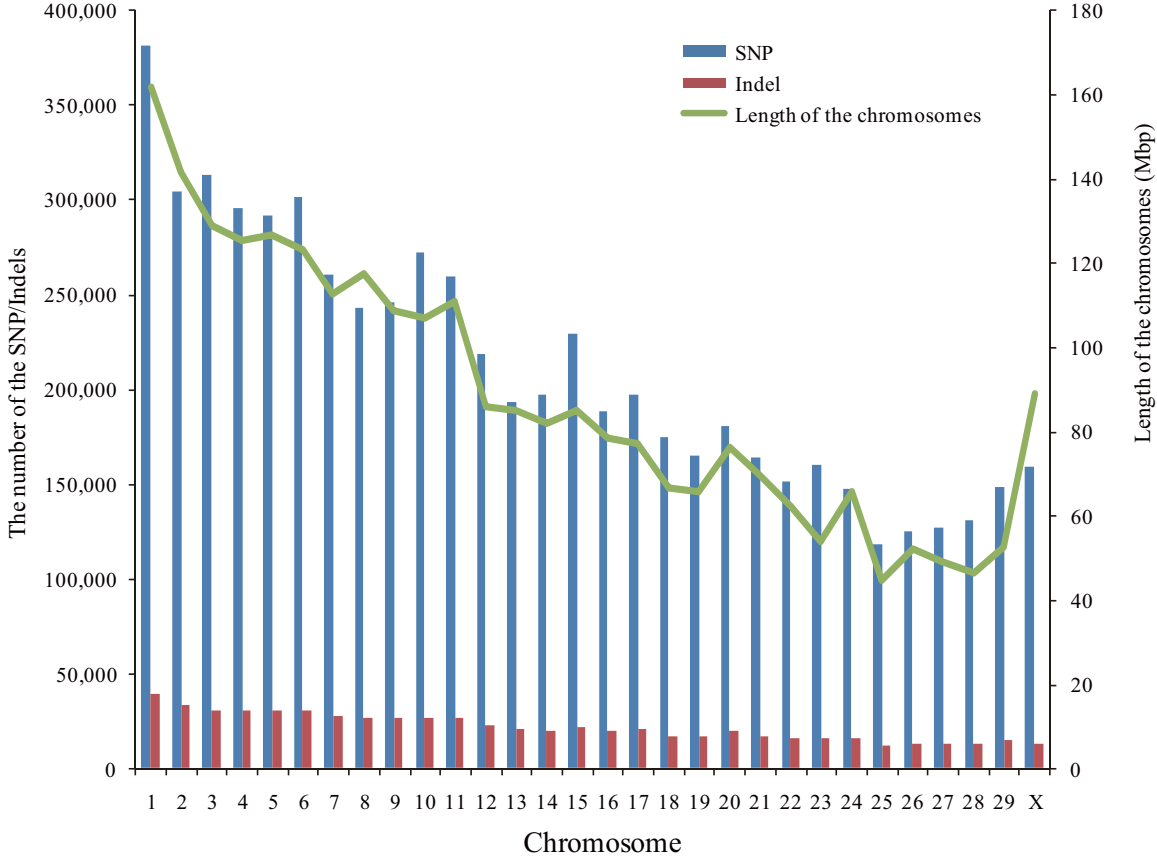

Supplement: Additional file 3 — The number of identified SNPs and indels for each chromosome. SNPs are shown in the blue columns, and indels are shown in the red columns. Length of chromosomes is indicated by the green line. [file 1471-2164-12-103-S3.PDF]

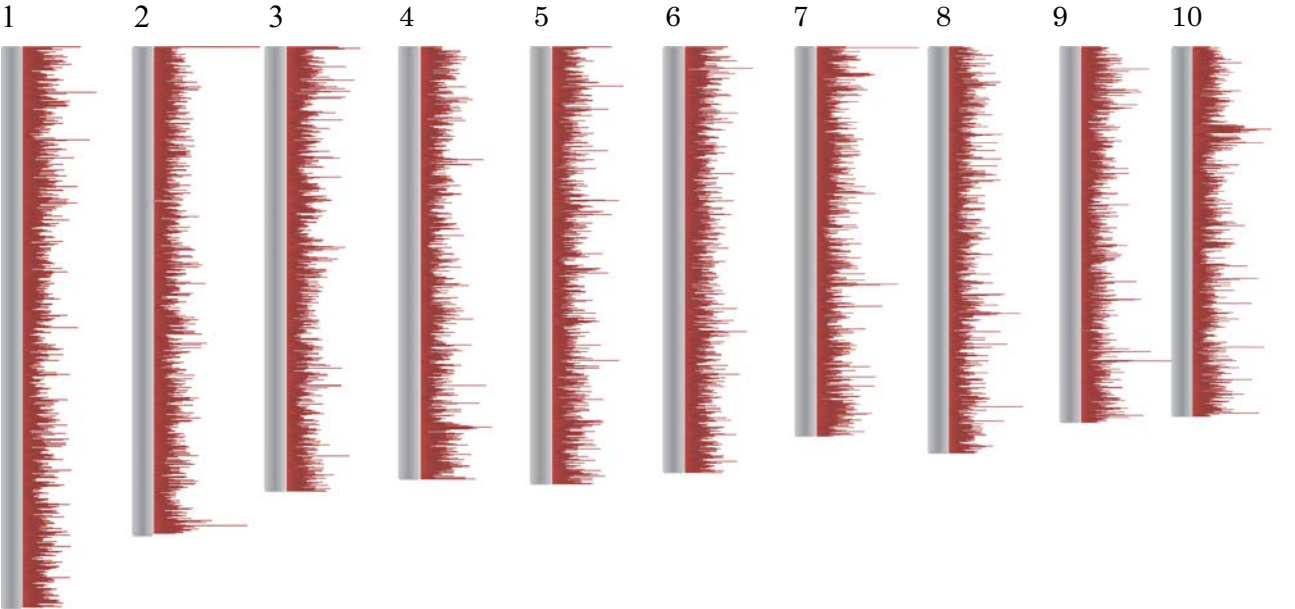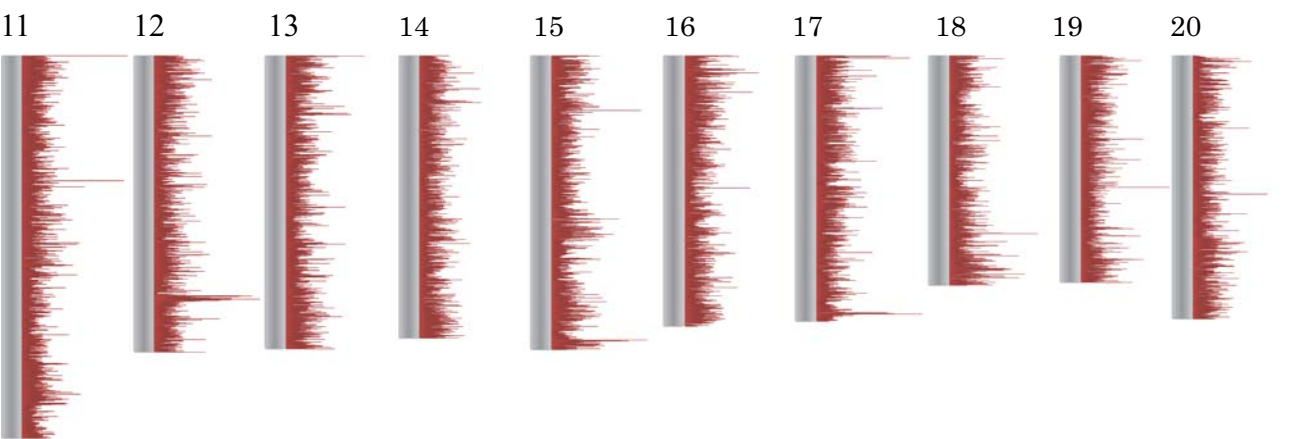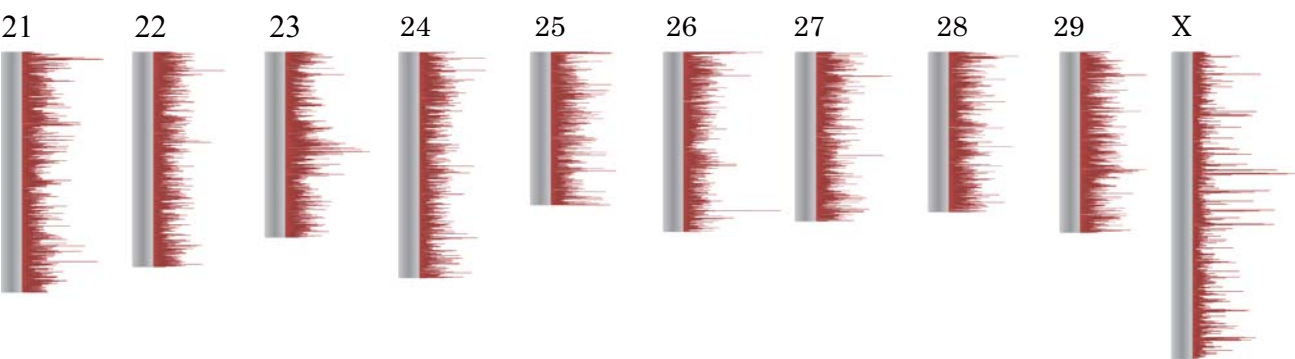

Supplement: Additional file 4 — SNP distribution on each chromosome. SNP density (SNPs per 1 kbp) is plotted by physical position. Relative length of the chromosomes was correlated with the length of each chromosome without repeat regions. [file 1471-2164-12-103-S4.PDF]

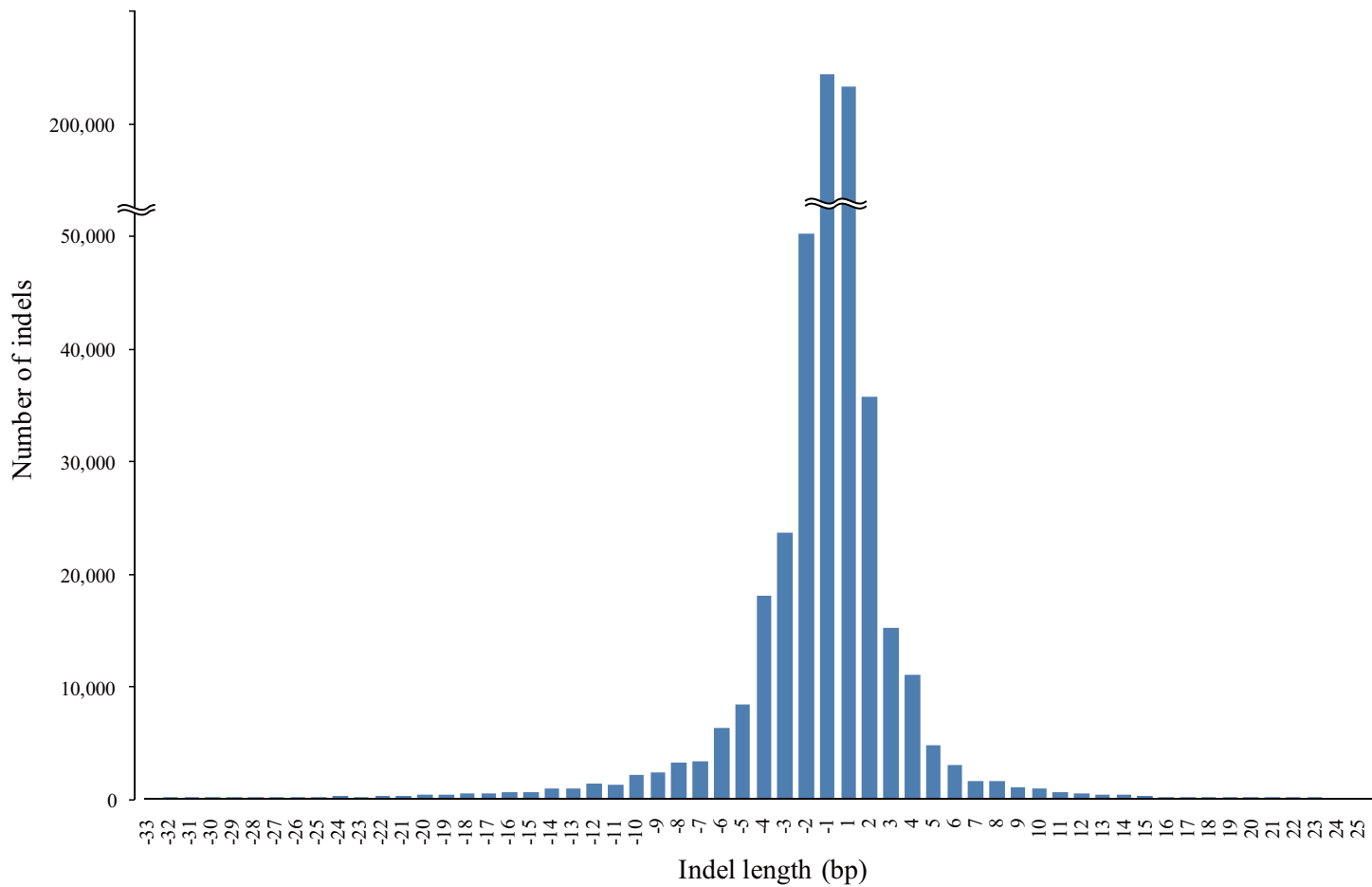

Supplement: Additional file 5 — Distribution of the size of indels. We identified 284,007 insertions (positive values) and 345,249 deletions (negative values). [file 1471-2164-12-103-S5.PDF]

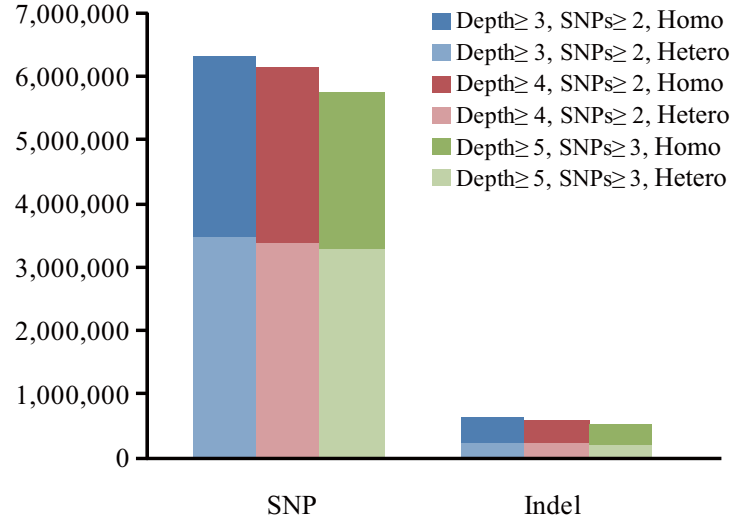

| Filter |      |              |           |       |         |       |  |
|--------|------|--------------|-----------|-------|---------|-------|--|
| Depth  | SNPs | mutation (%) | SNP       | %     | Indel   | %     |  |
| 3      | 2    | 30           | 6,303,790 | 100.0 | 629,256 | 100.0 |  |
| 4      | 2    | 30           | 6,154,201 | 97.6  | 601,920 | 95.7  |  |
| 5      | 3    | 30           | 5,762,560 | 91.4  | 546,613 | 86.9  |  |

Supplement: Additional file 9 — The number of SNPs and indels with various filters. Detected SNPs and indels were filtered with additional filters and the number of homozygous and heterozygous SNPs and indels was compared. Parameters for the filters were (1) Depth: the number of reads mapped to the SNP sites, (2) SNPs: the number of reads calling SNP at the SNP site, and (3) Mutation: the cutoff value of percent aligned reads calling the SNP per total mapped reads at the SNP sites. Cut off value was 30% in all filters. In the table, "%" means the reduced percentage of the number of SNPs/indels compared with basic parameters (i.e., Depth≥ 3, SNPs≥ 2, and Mutation≥ 30). [file 1471-2164-12-103-S9.PDF]
