# Supplementary material for: Whole-genome resequencing shows numerous genes with nonsynonymous SNPs in the Japanese native cattle Kuchinoshima-Ushi
Source: BMC Genomics. 2011 Feb 10;12:103. doi: 10.1186/1471-2164-12-103 (PMC3048544; doi:10.1186/1471-2164-12-103)
Supplement: Additional file 7 — GO terms which were over-represented in nsSNP-containing genes. [file 1471-2164-12-103-S7.DOC]

| Table S1. GO terms which were over-represented in nsSNP-containing genes. | | | | | |
| --- | --- | --- | --- | --- | --- |
| **Ontology** | **Description** | **Number among nsSNP including genes (107 genes)** | **Number among reference genes (14,927 genes)** | **p-value** | **q-value** *1 |
| Molecular function | Protein binding | 64 | 1881 | 3.4E-18 | 2.2E-16 |
| Molecular function | Transcription factor binding | 6 | 16 | 1.9E-09 | 1.2E-07 |
| Molecular function | Carbohydrate binding | 9 | 71 | 6.5E-09 | 4.1E-07 |
| Molecular function | Enzyme binding | 6 | 32 | 1.9E-07 | 0.000012 |
| Molecular function | Polysaccharide binding | 5 | 25 | 1.4E-06 | 0.00009 |
| Molecular function | Pattern binding | 5 | 25 | 1.4E-06 | 0.00009 |
| Molecular function | Sugar binding | 6 | 48 | 2.3E-06 | 0.00014 |
| Molecular function | Protein tyrosine kinase activity | 8 | 118 | 5.5E-06 | 0.00035 |
| Molecular function | Protein serine/threonine kinase activity | 7 | 100 | 0.000017 | 0.0011 |
| Molecular function | Protein dimerization activity | 5 | 69 | 0.00023 | 0.014 |
| Molecular function | Transferase activity | 23 | 1319 | 0.00072 | 0.045 |
| Molecular function | Binding | 90 | 7365 | 0.00072 | 0.046 |

*1 the value of the multiple-test (Bonferroni) adjusted p-value.
