# Supplementary material for: Whole-genome resequencing shows numerous genes with nonsynonymous SNPs in the Japanese native cattle Kuchinoshima-Ushi
Source: BMC Genomics. 2011 Feb 10;12:103. doi: 10.1186/1471-2164-12-103 (PMC3048544; doi:10.1186/1471-2164-12-103)
Supplement: Additional file 10 — List of species examined in this study. [file 1471-2164-12-103-S10.DOC]

| Table S2. List of species examined in this study. | | | |  |  |
| --- | --- | --- | --- | --- | --- |
| **Taxonomy** | | | | **Common name** | **Remarks** |
| **Subfamily** | **Tribe** | **Subtribe** | **Species** |  |  |
| Bovinae | Tragelaphini |  | *Taurotragus oryx* | Eland |  |
| Bovinae | Bovini | Bubalina | *Bubalus bubalis* | Murrah buffalo | Indian water buffalo |
| Bovinae | Bovini | Bubalina | *Bubalus bubalis* | Indian River buffalo | Indian water buffalo |
| Bovinae | Bovini | Bubalina | *Bubalus bubalis* | East Asian River buffalo | Asian water buffalo |
| Bovinae | Bovini | Bubalina | *Bubalus carabanesis* | Swamp buffalo | Asian water buffalo |
| Bovinae | Bovini | Bubalina | *Syncerus caffar* | Cape buffalo | African buffalo |
| Bovinae | Bovini | Bovina | *Bos taurus* | Holstein |  |
| Bovinae | Bovini | Bovina | *Bos taurus* | Hereford |  |
| Bovinae | Bovini | Bovina | *Bos taurus* | Tuli |  |
| Bovinae | Bovini | Bovina | *Bos taurus* | Kuchinoshima |  |
| Bovinae | Bovini | Bovina | *Bos javanicus* | Banteng |  |
| Bovinae | Bovini | Bovina | *Bos gaurus* | Gaur |  |
| Bovinae | Bovini | Bovina | *Bos grunniens* | Yak |  |
| Bovinae | Bovini | Bovina | *Bos frontalis* | Mithan (Gayal) | Domesticated gaur |
| Bovinae | Bovini | Bovina | *Bison bison* | Bison |  |
