# Supplementary material for: Whole-genome resequencing shows numerous genes with nonsynonymous SNPs in the Japanese native cattle Kuchinoshima-Ushi
Source: BMC Genomics. 2011 Feb 10;12:103. doi: 10.1186/1471-2164-12-103 (PMC3048544; doi:10.1186/1471-2164-12-103)
Supplement: Additional file 11 — Summary of the genes sequenced for the phylogenetic reconstruction. List of the genes and their accession numbers which were used for the phylogenetic analysis. [file 1471-2164-12-103-S11.DOC]

| Table S3. Summary of the genes sequenced for the phylogenetic reconstruction. | | | | |
| --- | --- | --- | --- | --- |
| **Common Name** | **Chromosome** | **Length (bp)** | **Description** | **Accession Numbers** |
| NRIP1 | 1 | 2332 | Nuclear factor RIP140 (Nuclear receptor interacting protein 1) | FI110745-FI110796 |
| PIT1 | 1 | 3451 | Pituitary-specific positive transcription factor 1 (Pit-1) (Growth hormone factor 1) (GHF-1) | FI110905-FI110982 |
| ITGBP5 | 1 | 2524 | Integrin beta-5 precursor | FI110410-FI110435, FI110479-FI110491, FI110498-FI110523 |
| IGFBP5 | 2 | 812 | Insulin-like growth factor binding protein 5 precursor (IGFBP-5) | FI110362-FI110385, FI110012-FI110013 |
| HFABP | 2 | 1741 | Fatty acid-binding protein, heart (H-FABP) (Heart-type fatty acid-binding protein) (Muscle fatty acid-binding protein) (M-FABP) (Mammary-derived growth inhibitor) (MDGI) | FI110292-FI110316, FI110329-FI110341, FI111009 |
| LACS3 | 2 | 2028 | Long-chain-fatty-acid-CoA ligase 3 (EC 6.2.1.3) (Long-chain acyl-CoA synthetase 3) (LACS 3) | FI110524-FI100575, FI110622-FI110634 |
| GMEB1 | 2 | 3389 | Glucocorticoid modulatory element binding protein 1 (GMEB-1) (DNA binding protein p96PIF) | FI110202-FI110240, FI110253-FI110291 |
| EGF | 6 | 618 | Epidermal growth factor (Urogastrone). | FI110064-FI110076 |
| ERA | 9 | 2367 | Estrogen receptor (ER) (Estradiol receptor) (ER-alpha) | FI110100-FI110131, FI110152-FI110176, FI110998-FI111001, FI111008 |
| MFGE8 | 21 | 2917 | Lactadherin precursor (Milk fat globule-EGF factor 8) (MFG-E8) (HMFG) (Breast epithelial antigen BA46) (MFGM) | FI110656-FI110681, FI110690-FI110715, FI110721-FI110733 |
